# A Modest Protective Effect of Thyrotropin against Bone Loss Is Associated with Plasma Triiodothyronine Levels

**DOI:** 10.1371/journal.pone.0145292

**Published:** 2015-12-17

**Authors:** Tae Hyuk Kim, Ji Young Joung, Mira Kang, Sun Kyu Choi, Kyunga Kim, Ju Young Jang, Yoon Young Cho, Yong-Ki Min, Jae Hoon Chung, Sun Wook Kim

**Affiliations:** 1 Division of Endocrinology & Metabolism, Department of Medicine, Samsung Medical Center, Sungkyunkwan University School of Medicine, Seoul, Korea; 2 Center for Health Promotion, Samsung Medical Center, Seoul, Korea; 3 Biostatistics and Clinical Epidemiology Center, Research Institute for Future Medicine, Samsung Medical Center, Sungkyunkwan University School of Medicine, Seoul, Korea; Baylor College of Medicine, UNITED STATES

## Abstract

**Background:**

The independent skeletal effect of thyrotropin (thyroid stimulating hormone, TSH) has been suggested in animal studies. However, clinical data on the association between bone loss and variations in TSH levels is inconsistent. This study aimed to investigate the relationship between TSH levels and bone mineral density (BMD).

**Methods:**

We conducted a cross-sectional study with 37,431 subjects (33,052 cases with euthyroidism and 4,379 cases with subclinical thyroid dysfunction) aged over 35 years. We performed thyroid function tests and measured BMD at the lumbar spine, femur neck, and total hip.

**Results:**

Levels of TSH and T3 were positively correlated in women (r = 0.076, P = 0.001) and uncorrelated in men. In both men and women, TSH levels correlated positively and T3 levels correlated negatively with BMD at all skeletal sites in age and body mass index adjusted analyses. BMD increased steadily with TSH levels from the subclinical hyperthyroid to subclinical hypothyroid range in subjects with T3 levels in the highest tertile (119.5–200.0 ng/dL), but was no longer significant in subjects with lower plasma T3 levels.

**Conclusions:**

The variations in TSH levels within the euthyroid and subclinical range were positively correlated with BMD in healthy men and women. The negative effect of T3 on BMD appears to be compensated for by increased TSH in subjects with plasma T3 levels in the upper normal range.

## Introduction

Overt hyperthyroidism is an established risk factor for low bone mineral density (BMD) [1]. Excess thyroid hormone promotes bone turnover, increasing the risk of trabecular perforation and fractures [2]. However, the relationship between bone loss and thyroid function is unclear in subjects with subclinical thyroid dysfunction and euthyroidism [3, 4].

Rodent model studies suggest that low or elevated thyrotropin (thyroid stimulating hormone, TSH) levels may play an important role in bone remodeling and homeostasis [5–8]. This independent skeletal effect of TSH may be clinically relevant in populations with biochemically defined subclinical thyroid dysfunction or euthyroidism, in which TSH levels are altered from 0.1 to 10.0 mIU/L but thyroid hormone levels are stable within the reference range. However, previous clinical studies have been unable to discern the effects of TSH and thyroid hormones on bone loss due to the well-known physiologic reciprocal relationship between TSH and free thyroxine (T4) in healthy subjects with intact hypothalamic-pituitary-thyroid axes [9]. Furthermore, many studies lacked statistical power due to an insufficient number of study participants or had limited generalizability as they focused on postmenopausal women.

In the present study, we sought to investigate the relationship between TSH and BMD in a large institutional cohort. We also examined whether plasma triiodothyronine (T3), the active form of thyroid hormone that may vary independently of TSH levels, affects the relationship between TSH and BMD.

## Materials and Methods

These cross-sectional analyses were based on the database of the institutional cohort of Health Promotion Center of the Samsung Medical Center in Seoul, Korea. From January 2007 to December 2013, 62,304 adults aged 35 years or older visited the center for routine health check-ups and underwent BMD and thyroid function tests (TFTs). After exclusion of 16 subjects due to invalid scans, we excluded 8,049 subjects with a history of cancer, diabetes mellitus, hyperlipidemia, or renal disease. We also excluded 3,995 subjects who were current smokers, as cigarette smoking is known to alter TSH level and bone metabolism [10, 11]. In addition, we excluded subjects with a history of thyroid disease, those on thyroxine or antithyroid drug treatment, and those taking medication that could interfere with thyroid function or bone metabolism (e.g., hormonal replacement, tamoxifen, bisphosphonate, calcium, or steroids). In total, 11,682 subjects were excluded according to these criteria. Among the remaining 38,562 subjects included in the final analysis, 33,052 (85.7%) subjects were euthyroid and 4,379 (11.4%) subjects had subclinical thyroid dysfunction. This study was approved by the Institutional Review Board of Samsung Medical Center and the need for informed consent was waived.

Each subject completed a health-related questionnaire addressing history of thyroid disease, other comorbidities, smoking habits and medication information at the visit. Current smoking was defined as smoking regularly for at least the past 12 months. Height and body weight were measured using a digital scale, and body mass index (BMI) was calculated. Blood samples were collected from each subject after an 8-hour overnight fast. The concentrations of TSH, total T3, and free T4 were measured at the central laboratory of Samsung Medical Center using an immunoradiometric assay kit (Beckman Coulter, Marseille, France) for measurement of TSH and radioimmunoassay kits (Beckman Coulter) for measurement of total T3 and free T4. Coefficients of variation were as follows: TSH, ≤3.7%; total T3, ≤6.3%; and free T4, ≤10.3%.

The subjects were placed into three groups based on the results of their TFTs [12]: 1) subclinical hyperthyroidism was defined as having TSH levels between 0.10 and 0.50 mIU/L with free T4 and total T3 concentrations in the reference range (free T4, 0.70–1.80 ng/dL; T3, 60.0–200.0 ng/dL), 2) euthyroid status was defined as having TSH levels between 0.51 and 5.00 mIU/L with free T4 and total T3 concentrations in the reference range; 3) subclinical hypothyroidism was defined as having TSH levels between 5.01 to 10.00 mIU/L with free T4 and total T3 concentrations in the reference range. Subjects with overt hyper- or hypothyroidism were excluded from the analyses.

BMD of the lumbar spine (L1-L4), femur neck, and total hip were measured via dual X-ray absorptiometry using Lunar Prodigy Advance (Madison, WI, USA) according to the manufacturer’s protocol. The left hip was scanned routinely and, in the case of a left hip fracture or device, the right hip was scanned. For all subjects in this study, BMD was measured with the same instruments. Data from our center showed that the coefficients of variation for duplicate measurement in 30 adults were 0.95%, 0.95%, and 1.35% at the lumbar spine, femur neck, and total hip, respectively.

We stratified subjects with subclinical thyroid dysfunction and euthyroid status. Data from women and men were analyzed separately. The values of TSH were normalized by base-10 logarithmic transformation or categorized as a group variable for further analyses. Possible associations among various parameters of TFT and BMD were tested using Pearson’s partial correlation by adjusting for age and BMI. In the euthyroid range of TSH level (0.51 to 5.00 mIU/L), a further subdivision was made into tertile categories so that all subjects were divided into five different TSH groups as follows [13]: subclinical hyperthyroid, 0.10–0.50 mIU/L; tertiles within the euthyroid range, 0.51–1.80, 1.81–2.85, and 2.86–5.00 mIU/L; and subclinical hypothyroid, 5.01–10.00 mIU/L.

Differences in BMD among TSH groups were identified by analysis of covariance (ANCOVA) by adjusting for age and BMI and expressed as estimated marginal means and SEM. Plasma T3 levels were grouped into tertiles as follows so that each group contains a third of the population: 119.5–200.0 ng/dL, 104.1–119.4 ng/dL, and 60.0–104.0 ng/dL. ANCOVA analyses with the covariates of age and BMI were used to examine whether TSH levels were related to BMD in each T3 group. Statistical analyses were performed using SAS version 9.4 (SAS Institute, Cary, NC, USA) software. A *P* value of <0.05 was considered statistically significant.

## Results

Overall, 37,431 adult subjects were analyzed for associations between thyroid function and BMD (Table 1). The mean age among subjects was 52.1 ± 9.2 years (50.1 ± 8.3 in women and 58.2 ± 9.2 in men). Among 28,300 women, 338 (1.2%) had subclinical hyperthyroidism, 3,291 (11.6%) had subclinical hypothyroidism, and 24,671 (87.2%) had euthyroidism. Among 9,131 men, 131 (1.4%) had subclinical hyperthyroidism, 619 (6.8%) had subclinical hypothyroidism, and 8,381 (91.8%) had euthyroidism. There were no significant differences in age or BMI according to thyroid status.

**Table 1:** Characteristics and BMD measurements of study subjects with subclinical thyroid dysfunction and euthyroidism.

	Women (n = 28,300)			Men (n = 9,131)		
	Subclinical		Subclinical	Subclinical		Subclinical
Characteristics	Hyperthyroid	Euthyroid	Hypothyroid	Hyperthyroid	Euthyroid	Hypothyroid
N	338	24 671	3291	131	8381	619
Age (y), mean (SD)	49.9 (8.7)	50.1 (8.3)	50.6 (8.3)	58.3 (9.2)	58.1 (9.2)	59.7 (9.6)
BMI (kg/m^2^), mean (SD)	22.7 (3.0)	22.5 (2.9)	22.7 (2.9)	24.2 (2.2)	24.5 (2.6)	24.4 (2.6)
TSH (mIU/L), median (IQR)	0.37 (0.25–0.45)	2.37 (1.62–3.27)	6.13 (5.49–7.18)	0.40 (0.29–0.45)	2.09 (1.43–2.94)	5.92 (5.40–7.05)
Free T4 (ng/dL), mean (SD)	1.30 (0.21)	1.20 (0.17)	1.16 (0.16)	1.31 (0.22)	1.27 (0.18)	1.20 (0.18)
Total T3 (ng/dL), mean (SD)	114.5 (22.0)	112.0 (18.5)	115.9 (19.4)	117.4 (20.7)	113.9 (18.8)	114.4 (18.4)
BMD (mg/cm^2^), mean (SD)						
Lumbar Spine	1117 (165)	1136 (165)	1139 (163)	1206 (178)	1215 (178)	1243 (193)
Femur Neck	884 (118)	892 (122)	894 (121)	936 (124)	947 (125)	961(133)
Total Hip	945 (122)	953 (126)	956 (124)	1019 (128)	1029 (132)	1041 (137)

From the correlation analysis adjusted for age and BMI, there was a robust negative relationship between TSH and free T4 level (r = -0.131 in women, r = -0.118 in men (all P <0.001; Table 2), whereas T3 and TSH correlated positively in women (r = 0.076, P <0.001) and did not correlate in men. The subgroup analysis confined to elderly subjects (>65 years), revealed a similar relationship between TSH and T3 (r = 0.051, P = 0.03 in women; r = -0.001, P = 0.95 in men) (S1 Table). In addition, the relationships between the total T3 and free T4 according to TSH categories within the highest T3 tertile were shown in S1 Fig. In both sexes, TSH correlated positively and T3 correlated negatively with BMD in all skeletal sites (Table 2). Free T4 was negatively associated with BMD measurements to a lesser degree in women and did not correlate in men.

**Table 2:** Correlation matrix between serum TSH, thyroid hormone concentrations, and BMD measurements^a^.

				Lumbar Spine	Femur Neck	Total Hip
	Log TSH	Free T4	Total T3	BMD	BMD	BMD
**Women**						
Log TSH (mIU/L)	1					
Free T4 (ng/dL)	-0.131^b^	1				
Total T3 (ng/dL)	0.076^b^	0.086^b^	1			
Lumbar Spine BMD (mg/cm^2^)	0.030^b^	-0.014^c^	-0.062^b^	1		
Femur Neck BMD (mg/cm^2^)	0.021^b^	-0.019^c^	-0.053^b^	0.615^b^	1	
Total Hip BMD (mg/cm^2^)	0.023^b^	-0.028^b^	-0.050^b^	0.647^b^	0.893^b^	1
**Men**						
Log TSH (mIU/L)	1					
Free T4 (ng/dL)	-0.118^b^	1				
Total T3 (ng/dL)	0.016	0.060^b^	1			
Lumbar Spine BMD (mg/cm^2^)	0.049^b^	-0.007	-0.038^b^	1		
Femur Neck BMD (mg/cm^2^)	0.047^b^	-0.010	-0.037^b^	0.604^b^	1	
Total Hip BMD (mg/cm^2^)	0.040^b^	-0.017	-0.029^c^	0.643^b^	0.893^b^	1

Abbreviation: Log TSH, logarithmic transformation of TSH concentration.

^a^ Values are partial correlation coefficients adjusted by age and BMI.

^b^ Correlation is significant at the 0.001 level (2-tailed).

^c^ Correlation is significant at the 0.05 level (2-tailed).

**Fig 1:**
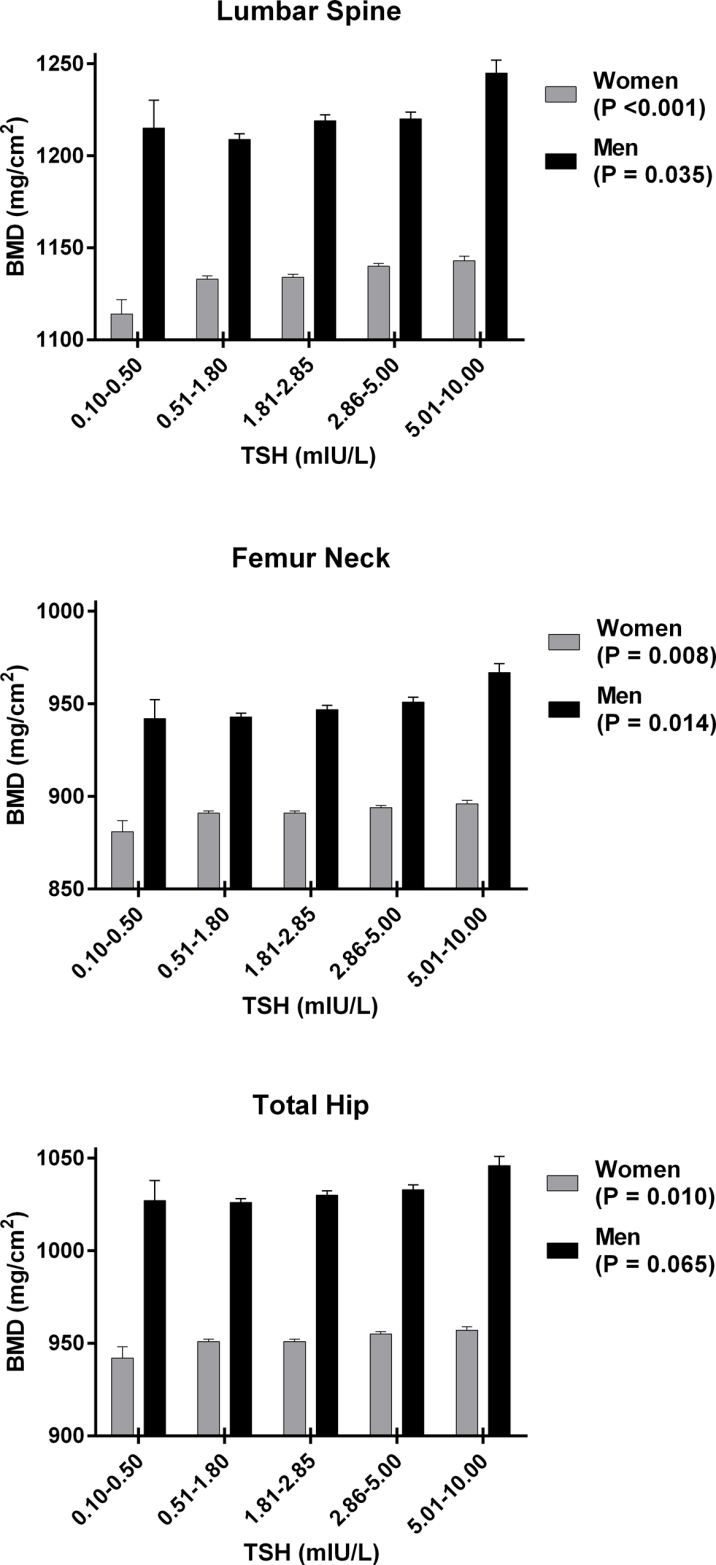
The effect of serum TSH concentration on BMD measurements. Data are shown as estimated marginal mean and SEM (error bar), with age and BMI used as covariates. P values for trends across strata of TSH were obtained using ANCOVA.

To examine the independent dose-response relationship between BMD and TSH level, we constructed ANCOVA models with age and BMI as covariates [14]. The changes in BMD compared to the changes in TSH across the subclinical range were generally modest at all skeletal sites (Fig 1). In women, BMD at all skeletal sites increased linearly with TSH from subclinical hyperthyroid to subclinical hypothyroid range. In men, lumbar spine and femur neck BMD increased linearly with TSH. Total hip BMD also tended to be elevated in subjects with increased TSH, although it did not reach statistical significance (P = 0.065).

**Fig 2:**
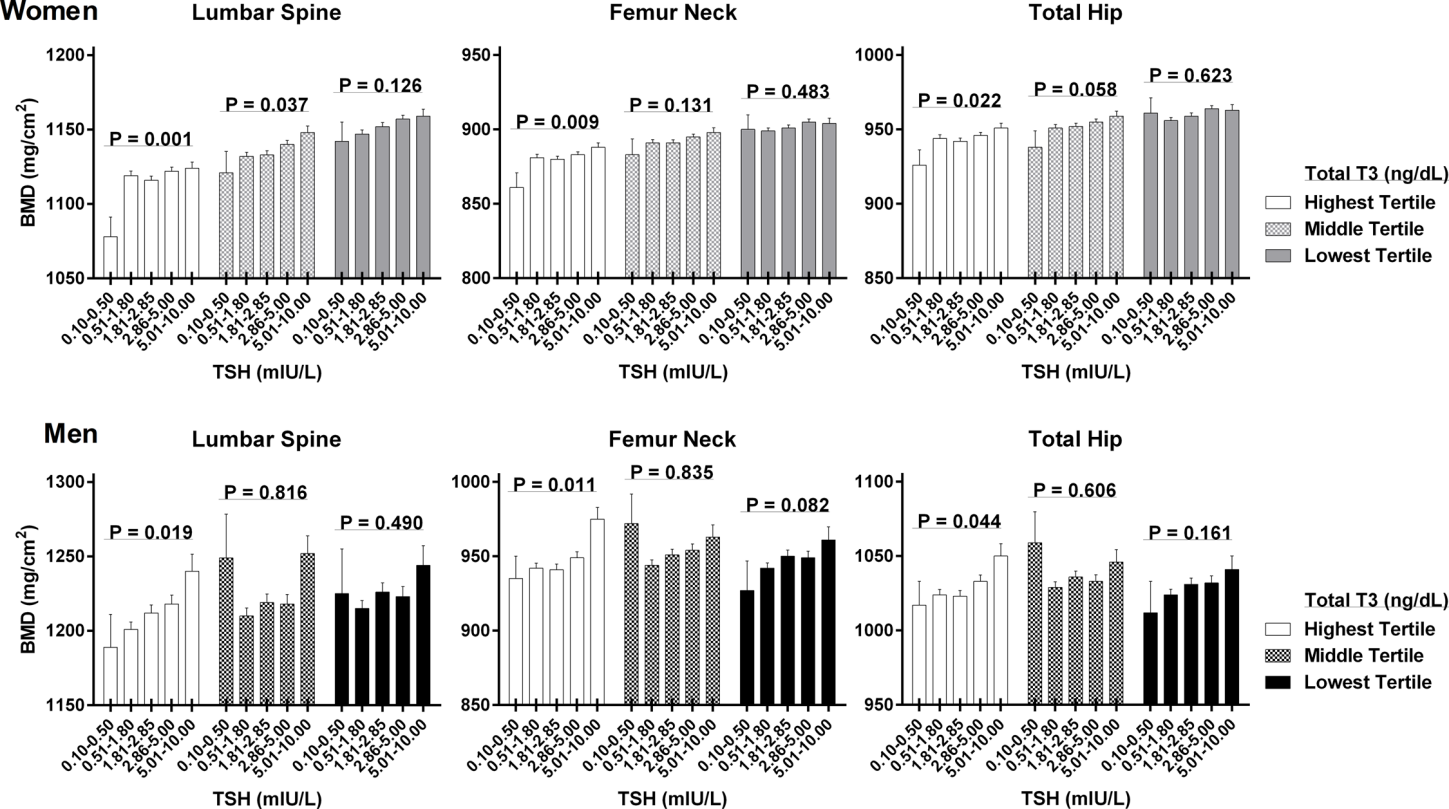
The effect of serum TSH concentration on BMD measurements according to total T3 concentration. Data are shown as estimated marginal mean and SEM (error bar), with age and BMI used as covariates. The shading patterns indicate tertile groups according to total T3 concentrations. P values for trends across strata of TSH were obtained by ANCOVA.

Differences in BMD between TSH groups were evident after classifying subjects according to plasma T3 levels. In women, significant positive relationships between TSH and BMD at all skeletal sites were observed when T3 levels were in the highest tertile (119.5–200.0 ng/dL) (Fig 2). In this group, the differences in BMD among subjects with subclinical hyperthyroidism and subclinical hypothyroidism were 46, 27, and 25 mg/cm^2^ at the lumbar spine, femur neck, and total hip, respectively. However, there was no significant relationship between TSH and BMD in the group with T3 levels in the middle and lowest tertiles (104.1–119.4 ng/dL and 60.0–104.0 ng/dL respectively), except for BMD at lumbar spine in those with T3 levels in the middle tertile (P = 0.037). In men, significant positive relationships between TSH and BMD at all sites were observed only in groups with T3 levels in the highest tertile. In subjects with the highest tertile of T3 levels, the differences in BMD between subjects with subclinical hyperthyroidism and subclinical hypothyroidism were 51, 40, and 33 mg/cm^2^ at the lumbar spine, femur neck, and total hip, respectively.

## Discussion

In this large cross-sectional study, we demonstrated that variations in TSH levels within subclinical range were related to BMD in a healthy adult population with normal thyroid hormone levels. Lower TSH and higher T3 levels were associated with reduced BMD at all skeletal sites in both sexes. The negative impact of T3 on BMD appeared to be compensated for by increased TSH only in subjects with T3 levels in the upper normal range.

To the best of our knowledge, this is the first clinical study to show the protective effect of TSH on bone loss independent of the effects of active thyroid hormone, T3. Based on the distinct distribution between plasma T3 and TSH levels unrelated to simple feedback inhibitory mechanisms, we performed stratified analysis to explore the relationships between TSH, T3 and BMD in a large population of healthy men and women. We excluded patients with various chronic conditions and medication history that may act as confounders in the relationship between thyroid function and bone mass. Clinicians should consider the modest protective effect of TSH against bone loss when treating biochemically defined subclinical hyper- or hypothyroidism, particularly when it is in combination with upper normal range plasma T3 levels.

Plasma levels of active thyroid hormone T3 are remarkably stable in healthy adult individuals [15] as a result of combined homeostatic mechanisms by the hypothalamic-pituitary-thyroid axis and a group of deiodinases found in extrathyroidal tissues, including the skeleton [16, 17]. Plasma T3 equilibrates rapidly in most tissues. In bone, T3 mainly acts on osteoblasts to increase bone turnover in which resorption outpaces formation [18]. Interestingly, a recent study of a human osteoblast cell line showed increased expression of deiodinase 2 (which converts prohormone T4 to T3) by administration of TSH [19], suggesting that TSH may indirectly promote bone turnover by increasing local T3 availability in osteoblasts in addition to its direct action on bone remodeling [20]. When cellular T3 levels are sufficient, deiodinase 2 activities are repressed [21] and the direct action of TSH as a negative regulator of bone remodeling dominates.

Previous studies investigating the risk of bone loss and fractures in combination with abnormal TSH levels have been inconsistent. In a study of postmenopausal women, TSH levels above the reference range were associated with a 35% reduced risk of nonvertebral fractures, although TSH was not related to statistically significant BMD change [9]. Conversely, an Israeli study reported that TSH levels within the lower normal range were associated with an increased risk of hip fractures in elderly women, but not in men [22]. However, Lin et al found no relationship between TSH and BMD under normal thyroid conditions in a Chinese population [23] and a recent cross-sectional analysis of the Cardiovascular Health Study concluded that subclinical hyperthyroidism was not associated with BMD loss in either the hip or spine in older men and women [3]. Similarly, a study-level meta-analysis of seven population-based cohorts did not find an association between subclinical hyperthyroidism and risk of fractures, but the interpretation was limited by the heterogeneity of the studies included [24]. In the midst of uncertainty, a recent meta-analysis analyzing the individual data of 13 past studies involving 70,298 participants found a significantly elevated risk of hip and other fractures in patients with subclinical hyperthyroidism, particularly those with TSH levels less than 0.10 mIU/L [25].

Given that TSH can either indirectly promote or directly suppress bone turnover according to local T3 availability, it is not surprising that previous clinical studies, which did not take into account plasma T3 status, have produced inconsistent results regarding the effect of TSH on bone remodeling. We observed a significant dose-dependent trend between TSH levels and BMD exclusively in men and women in the highest tertile T3 level. The findings from our large population study are consistent with basic mechanistic studies as mentioned above.

There are several limitations to this study. This was a cross-sectional observational study, and therefore the causal relationship between TSH and T3 and BMD cannot be affirmed by this data. We cannot address whether the treatment of subclinical thyroid dysfunction could lead to reciprocal changes in BMD, although nonrandomized trials have suggested that normalization of suppressed TSH increases BMD in postmenopausal women with subclinical hyperthyroidism [26, 27]. Information for markers of bone formation and resorption was not assessed. The stratified analysis for men with subclinical hyperthyroidism was limited due to the small number of subjects in the category (0.3% of study population). Lastly, our study subjects were of medium to high socioeconomic status, which could contribute to selection bias, although data regarding the relationship between socioeconomic status and BMD are conflicting [28].

In conclusion, in a large cohort of health-screening recipients, we demonstrated that variations in TSH levels even within the subclinical range were positively correlated with BMD in healthy men and women with normal thyroid hormone levels. Furthermore, plasma T3 status may affect the relationship between TSH and BMD. Thus the protective effect of TSH against bone loss should be taken into consideration during treatment decisions regarding subclinical thyroid dysfunction, particularly when it presents in combination with upper normal range plasma T3 levels.

## Supporting Information

S1 Fig Distribution of total T3 and free T4 concentrations according to TSH categories in subjects with the highest T3 tertile (119.5–200.0 ng/dL). Data are shown as mean and SEM (error bar). P values for trends across strata of TSH were obtained using ANOVA.
(TIF)

S1 Table Correlation matrix between serum TSH, thyroid hormone concentrations, and BMD measurements in subjects older than 65 years. (DOC)

## References

[1] Lee MS, Kim SY, Lee MC, Cho BY, Lee HK, Koh CS, et al Negative correlation between the change in bone mineral density and serum osteocalcin in patients with hyperthyroidism. J Clin Endocrinol Metab. 1990;70:766–770. 2307730 10.1210/jcem-70-3-766

[2] Mosekilde L, Eriksen EF, Charles P. Effects of thyroid hormones on bone and mineral metabolism. Endocrinol Metab Clin North Am. 1990;19:35–63. 2192868

[3] Garin MC, Arnold AM, Lee JS, Robbins J, Cappola AR. Subclinical thyroid dysfunction and hip fracture and bone mineral density in older adults: the cardiovascular health study. J Clin Endocrinol Metab. 2014;99:2657–2664. 10.1210/jc.2014-1051 24878045 PMC4121038

[4] Faber J, Galloe AM. Changes in bone mass during prolonged subclinical hyperthyroidism due to L-thyroxine treatment: a meta-analysis. Eur J Endocrinol. 1994;130:350–356. 8162163 10.1530/eje.0.1300350

[5] Baliram R, Sun L, Cao J, Li J, Latif R, Huber AK, et al Hyperthyroid-associated osteoporosis is exacerbated by the loss of TSH signaling. J Clin Invest. 2012;122:3737–3741. 10.1172/JCI63948 22996689 PMC3461920

[6] Sun L, Vukicevic S, Baliram R, Yang G, Sendak R, McPherson J, et al Intermittent recombinant TSH injections prevent ovariectomy-induced bone loss. Proc Natl Acad Sci U S A. 2008;105:4289–4294. 10.1073/pnas.0712395105 18332426 PMC2393772

[7] Sampath TK, Simic P, Sendak R, Draca N, Bowe AE, O'Brien S, et al Thyroid-stimulating hormone restores bone volume, microarchitecture, and strength in aged ovariectomized rats. J Bone Miner Res. 2007;22:849–859. 17352644 10.1359/jbmr.070302

[8] Abe E, Marians RC, Yu W, Wu XB, Ando T, Li Y, et al TSH is a negative regulator of skeletal remodeling. Cell. 2003;115:151–162. 14567913 10.1016/s0092-8674(03)00771-2

[9] Murphy E, Gluer CC, Reid DM, Felsenberg D, Roux C, Eastell R, et al Thyroid function within the upper normal range is associated with reduced bone mineral density and an increased risk of nonvertebral fractures in healthy euthyroid postmenopausal women. J Clin Endocrinol Metab. 2010;95:3173–3181. 10.1210/jc.2009-2630 20410228

[10] Asvold BO, Bjoro T, Nilsen TI, Vatten LJ. Tobacco smoking and thyroid function: a population-based study. Arch Intern Med. 2007;167:1428–1432. 17620538 10.1001/archinte.167.13.1428

[11] Law MR, Hackshaw AK. A meta-analysis of cigarette smoking, bone mineral density and risk of hip fracture: recognition of a major effect. BMJ. 1997;315:841–846. 9353503 10.1136/bmj.315.7112.841 PMC2127590

[12] Kim TH, Kim KW, Ahn HY, Choi HS, Won H, Choi Y, et al Effect of seasonal changes on the transition between subclinical hypothyroid and euthyroid status. J Clin Endocrinol Metab. 2013;98:3420–3429. 10.1210/jc.2013-1607 23771919

[13] Grimnes G, Emaus N, Joakimsen RM, Figenschau Y, Jorde R. The relationship between serum TSH and bone mineral density in men and postmenopausal women: the Tromso study. Thyroid. 2008;18:1147–1155. 10.1089/thy.2008.0158 18925834

[14] Kim KM, Choi SH, Lim S, Moon JH, Kim JH, Kim SW, et al Interactions between dietary calcium intake and bone mineral density or bone geometry in a low calcium intake population (KNHANES IV 2008–2010). J Clin Endocrinol Metab. 2014;99:2409–2417. 10.1210/jc.2014-1006 24684465

[15] Abdalla SM, Bianco AC. Defending plasma T3 is a biological priority. Clin Endocrinol (Oxf). 2014;81:633–641. 25040645 10.1111/cen.12538 PMC4699302

[16] Bassett JH, Boyde A, Howell PG, Bassett RH, Galliford TM, Archanco M, et al Optimal bone strength and mineralization requires the type 2 iodothyronine deiodinase in osteoblasts. Proc Natl Acad Sci U S A. 2010;107:7604–7609. 10.1073/pnas.0911346107 20368437 PMC2867713

[17] Bianco AC, Kim BW. Deiodinases: implications of the local control of thyroid hormone action. J Clin Invest. 2006;116:2571–2579. 17016550 10.1172/JCI29812 PMC1578599

[18] Mosekilde L, Melsen F. Effect of antithyroid treatment on calcium-phosphorus metabolism in hyperthyroidism. II: Bone histomorphometry. Acta Endocrinol (Copenh). 1978;87:751–758. 580521 10.1530/acta.0.0870751

[19] Morimura T, Tsunekawa K, Kasahara T, Seki K, Ogiwara T, Mori M, et al Expression of type 2 iodothyronine deiodinase in human osteoblast is stimulated by thyrotropin. Endocrinology. 2005;146:2077–2084. 15650076 10.1210/en.2004-1432

[20] de Lloyd A, Bursell J, Gregory JW, Rees DA, Ludgate M. TSH receptor activation and body composition. J Endocrinol. 2010;204:13–20. 10.1677/JOE-09-0262 19759194

[21] Bianco AC, Salvatore D, Gereben B, Berry MJ, Larsen PR. Biochemistry, cellular and molecular biology, and physiological roles of the iodothyronine selenodeiodinases. Endocr Rev. 2002;23:38–89. 11844744 10.1210/edrv.23.1.0455

[22] Leader A, Ayzenfeld RH, Lishner M, Cohen E, Segev D, Hermoni D. Thyrotropin levels within the lower normal range are associated with an increased risk of hip fractures in euthyroid women, but not men, over the age of 65 years. J Clin Endocrinol Metab. 2014;99:2665–2673. 10.1210/jc.2013-2474 24885627

[23] Lin JD, Pei D, Hsia TL, Wu CZ, Wang K, Chang YL, et al The relationship between thyroid function and bone mineral density in euthyroid healthy subjects in Taiwan. Endocr Res. 2011;36:1–8. 10.3109/07435800.2010.514877 21226562

[24] Wirth CD, Blum MR, da Costa BR, Baumgartner C, Collet TH, Medici M, et al Subclinical thyroid dysfunction and the risk for fractures: a systematic review and meta-analysis. Ann Intern Med. 2014;161:189–199. 10.7326/M14-0125 25089863 PMC4403766

[25] Blum MR, Bauer DC, Collet TH, Fink HA, Cappola AR, da Costa BR, et al Subclinical Thyroid Dysfunction and Fracture Risk: A Meta-analysis. JAMA. 2015;313:2055–2065. 10.1001/jama.2015.5161 26010634 PMC4729304

[26] Faber J, Jensen IW, Petersen L, Nygaard B, Hegedus L, Siersbaek-Nielsen K. Normalization of serum thyrotrophin by means of radioiodine treatment in subclinical hyperthyroidism: effect on bone loss in postmenopausal women. Clin Endocrinol (Oxf). 1998;48:285–290. 9578817 10.1046/j.1365-2265.1998.00427.x

[27] Mudde AH, Houben AJ, Nieuwenhuijzen Kruseman AC. Bone metabolism during anti-thyroid drug treatment of endogenous subclinical hyperthyroidism. Clin Endocrinol (Oxf). 1994;41:421–424. 7955452 10.1111/j.1365-2265.1994.tb02571.x

[28] Brennan SL, Henry MJ, Wluka AE, Nicholson GC, Kotowicz MA, Williams JW, et al BMD in population-based adult women is associated with socioeconomic status. J Bone Miner Res. 2009;24:809–815. 10.1359/jbmr.081243 19113909

