# Supplementary material for: A Modest Protective Effect of Thyrotropin against Bone Loss Is Associated with Plasma Triiodothyronine Levels
Source: PLoS One. 2015 Dec 17;10(12):e0145292. doi: 10.1371/journal.pone.0145292 (PMC4682993; doi:10.1371/journal.pone.0145292)
Supplement: S1 Table — (DOC) [file pone.0145292.s002.doc]

**S1 Table. Correlation matrix between serum TSH, thyroid hormone concentrations, and BMD measurements in subjects older than 65 years***^a^*

|  |  |  |  | **Lumbar Spine** | **Femur Neck** | **Total Hip** |
| --- | --- | --- | --- | --- | --- | --- |
|  | **Log TSH** | **Free T4** | **Total T3** | **BMD** | **BMD** | **BMD** |
| **Women (n = 1773)** |  |  |  |  |  |  |
| Log TSH (mIU/L) | 1 |  |  |  |  |  |
| Free T4 (ng/dL) | -0.159^b^ | 1 |  |  |  |  |
| Total T3 (ng/dL) | 0.051^c^ | 0.058^c^ | 1 |  |  |  |
| Lumbar Spine BMD (mg/cm^2^) | 0.088^b^ | 0.040 | 0.009 | 1 |  |  |
| Femur Neck BMD (mg/cm^2^) | 0.063^c^ | 0.037 | -0.008 | 0.570^b^ | 1 |  |
| Total Hip BMD (mg/cm^2^) | 0.068^c^ | 0.015 | 0.006 | 0.602^b^ | 0.873^b^ | 1 |
|  |  |  |  |  |  |  |
| **Men (n = 2406)** |  |  |  |  |  |  |
| Log TSH (mIU/L) | 1 |  |  |  |  |  |
| Free T4 (ng/dL) | -0.148^b^ | 1 |  |  |  |  |
| Total T3 (ng/dL) | -0.001 | 0.065^c^ | 1 |  |  |  |
| Lumbar Spine BMD (mg/cm^2^) | 0.057^c^ | 0.011 | -0.044^c^ | 1 |  |  |
| Femur Neck BMD (mg/cm^2^) | 0.072^b^ | -0.025 | -0.042^c^ | 0.591^b^ | 1 |  |
| Total Hip BMD (mg/cm^2^) | 0.067^c^ | -0.028 | -0.042^c^ | 0.645^b^ | 0.892^b^ | 1 |

Abbreviation: Log TSH, logarithmic transformation of TSH concentration.

^a^ Values are partial correlation coefficients adjusted by age and BMI.

^b^ Correlation is significant at the 0.001 level (2-tailed).

^c^ Correlation is significant at the 0.05 level (2-tailed).

**Supplemental Figure Legend**

**S1 Fig.** Distribution of total T3 and free T4 concentrations according to TSH categories in subjects with the highest T3 tertile (119.5-200.0 ng/dL). Data are shown as mean and SEM (error bar). P values for trends across strata of TSH were obtained using ANOVA.
